# Association of Adiponectin Gene Polymorphism rs266729 in Obese People with Type Two Diabetes Mellitus in North Bulgaria’s Population

**DOI:** 10.3390/ijms27052359

**Published:** 2026-03-03

**Authors:** Tihomir Rashev, Stefan Trifonov

**Affiliations:** Department of Anatomy, Histology, Cytology and Biology, Medical University—Pleven, 5800 Pleven, Bulgaria

**Keywords:** adiponectin, polymorphism, obesity, type 2 diabetes mellitus

## Abstract

The alarming surge in obesity rates represents a critical health concern in developed nations. This condition is associated with several health issues grouped under the term metabolic syndrome. This alarming data mandates an investigation into the causes and risk of this syndrome. One category of causes is genetic factors. The adiponectin gene has been subject to several studies exploring its single nucleotide polymorphisms, especially rs266729, across diverse populations. This study included 156 subjects. Body weight, body mass index, and blood pressure were measured, and levels of fasting plasma glucose (FPG), total cholesterol, triglycerides, HDL, and LDL were determined. Genotyping was performed. There was significant association of rs266729 polymorphism with obesity and type 2 diabetes mellitus (T2DM), with notably higher frequency of the G allele among these subjects. Additionally, the results demonstrated significant influence on the detected levels of HDL. Individuals carrying the homozygous (GG) and heterozygous (CG) genotypes exhibited a three-fold and two-fold increased risk of elevated FPG levels, respectively. There was also a significant association of these genotypes with T2DM when compared with the wild-type genotype (CC). These observations strongly indicated a potential involvement of the adiponectin gene polymorphism rs266729 in the pathogenesis of T2DM among subjects from North Bulgaria.

## 1. Introduction

The alarming surge in obesity rates represents a critical health concern in developed nations. This condition is intricately associated with an array of health issues grouped under the term “metabolic syndrome”, encompassing the development of insulin resistance, type 2 diabetes mellitus (T2DM), cardiovascular complications, and fatty liver disease [1]. The intricate interplay between obesity and T2DM stems from their shared link with insulin resistance, a key factor in their pathogenesis [1]. At the forefront of health challenges in the 21st century, T2DM constitutes a substantial majority (90–95%) of diabetes cases. It spans a spectrum from predominantly insulin resistance with relative insulin deficiency to scenarios characterized by an insulin secretory defect alongside insulin resistance [2]. Presently afflicting a staggering 317 million individuals worldwide, in Bulgaria alone it impacts around 9.9% of the adult population, totaling approximately 519,300 individuals [3]. This escalating prevalence correlates with the rapid upsurge in obesity rates witnessed across various countries [4]. The concerning data mandates a comprehensive investigation of the causes and risks associated with metabolic syndrome and specifically T2DM. One category of these causes includes genetic factors. Scientific advancements, such as the development of genome-wide association studies, have enabled the identification of several genes associated with the risk for development of T2DM. Among them, the adiponectin gene has been subject to several studies exploring its single nucleotide polymorphisms (SNPs) across diverse populations.

Obesity is characterized by an augmented storage of fatty acids and an enlarged mass of adipose tissue within the body. Adipose tissue evolved from its initial perception solely as a site for storing triacylglycerols. Over recent decades, significant accumulation of experimental data has enriched our understanding of the intricate biology and biochemistry of adipose tissue [5,6,7]. This once-considered inert fat storage depot is now acknowledged as a metabolically dynamic organ. Not only does it store excess energy, but it also functions as an endocrine organ synthesizing a variety of biologically active compounds crucial in regulating metabolic equilibrium. This dynamic tissue comprises not only adipocytes but also a diverse array of cell types within the stromal vascular fraction like blood cells, endothelial cells, pericytes, adipose precursor cells, and others [6,7,8,9]. Research has unveiled the non-uniform nature of adipose tissue, demonstrating variations based on body location, with respect to its capacity to secrete adipocytokines, cellular composition, phenotype diversity, and quantity and ratio of constituent components like adipocytes, blood vessel stromal cells, and immune cells [10]. The evolving recognition of adipose tissue as a vital organ underscores its integral role within a complex network governing diverse biological functions [11]. White adipose tissue stands as a potential contender for the largest endocrine tissue in humans, and the pleiotropic functions of its cells are capable of secreting a myriad of hormones, growth factors, enzymes, cytokines, complement factors, and matrix proteins. These adipocytes also express receptors for numerous factors crucial in regulating multiple physiological processes, including food intake, energy expenditure, metabolic equilibrium, immune responses, and blood pressure regulation [12,13]. Functionally, adipose tissue actively regulates cellular functions through an intricate web of endocrine, paracrine, and autocrine signaling. This complex signaling network exerts significant influences on various tissues, including the hypothalamus, pancreas, liver, skeletal muscle, kidneys, endothelium, and immune system, portraying white adipose tissue as a remarkably active endocrine organ [9]. Notably, experimental data reveals distinctive differences in adipocytokine synthesis and secretion between visceral fat and subcutaneous adipose tissue, with visceral fat displaying heightened activity. Both types exhibit unique adipocytokine profiles, characterized by differential concentrations of specific markers. For instance, visceral tissue demonstrates elevated levels of interleukin-6 and plasminogen activator inhibitor 1 (PAI-1), whereas subcutaneous adipose tissue displays increased concentrations of leptin and adiponectin [10]. The secretion of bioactive substances, such as adipocytokines in obese individuals, may contribute to various aspects of the metabolic syndrome. An imbalance in the production of pro- and anti-inflammatory adipocytokines, exemplified by oversecretion of potentially harmful ones like PAI-1, tumor necrosis factor-α, or visfatin and reduced secretion of beneficial adipocytokines like adiponectin, might underline lifestyle-related diseases including T2DM, hyperlipidemia, hypertension, and atherosclerosis, collectively constituting the metabolic syndrome [13,14]. Controlling key adipocytokines, such as adiponectin, presents a potential avenue for efficient therapeutic intervention, warranting careful exploration [15].

Recent studies have elucidated the robust impact of obesity (BMI ≥ 25 kg/m^2^) on adipocytokine secretion and its correlation with insulin resistance [16,17,18,19]. The adiponectin gene (*AdipoQ*), located on chromosome 3q27 and structurally related to the complement 1q family, was identified in 1995. Structurally, adiponectin consists of a *C*-terminal globular domain and an *N*-terminal collagenous domain, sharing extensive sequence homology with collagen VIII and X [20]. Adiponectin exists as a full-length protein of 30 kDa and circulates in three distinct isoforms: a low-molecular weight trimer (LMW), a medium-molecular weight hexamer (MMW, composed of a trimer-dimer), and a high-molecular weight multimeric isoform (HMW) [21]. Notably, a fragment containing the globular domain of adiponectin has exhibited potent metabolic effects in multiple tissues. Exclusive secretion of adiponectin occurs from adipose tissue cells [22]. Human plasma adiponectin levels show a robust negative correlation with adipose tissue quantity, often resulting in lower levels in obese individuals [23,24]. In cases of T2DM, plasma adiponectin levels are generally diminished [25]. This decrease might play a contributory role in the onset of insulin resistance and T2DM [26]. The association of adiponectin with T2DM primarily relates to a decline in the circulating HMW isoform, while the levels of other oligomeric forms remain relatively unchanged. The regulation of circulating adiponectin oligomers primarily occurs during their release from adipocytes, with minimal interconversion between isoforms post-release [1]. In various obesity models (genetic or diet-induced) adiponectin has demonstrated an ability to enhance whole-body insulin sensitivity [27]. Functionally, adiponectin engages three receptors—AdipoR1, AdipoR2, and T-cadherin. Activation of AdipoR1 and R2 induces increased β-oxidation of fatty acids in hepatic and skeletal muscle tissues, heightened lactate production in skeletal muscle, decreased hepatic gluconeogenesis, elevated cellular glucose uptake, and inhibition of oxidative stress and inflammation [25]. Adiponectin’s role in regulating energy expenditure occurs via AMP-activated protein kinase (AMPK) activation in the hypothalamus, where AdipoR1 and R2 co-localize with the leptin receptor [28]. Studies have demonstrated that adiponectin stimulates appetite while reducing energy expenditure, with these effects being nullified upon AdipoR1 ablation or AMPK signaling inhibition [29].

According to the National Center for Biotechnology Information (NCBI), the adiponectin locus in humans encompasses 683 single nucleotide polymorphisms (SNPs), with 33 of these SNPs cited in PubMed [30]. Among these, rs266729, a common genetic variant situated in the promoter region of *AdipoQ*, exhibits a C>G substitution at the 11377 position. This variant has been associated with T2DM in Asian populations [30,31]. Thus, this study is important because it is one of the few that investigates the frequency and correlation of this SNP in Northern Bulgarians with obesity and T2DM.

## 2. Results

A total of 156 individuals were included in the study. Among them, 75 individuals were male and 81 were female. The age of the participants in the study was in the range of 35 to 85 years. All subjects were distributed into two groups based on their body mass index (BMI): target group (obese with BMI ≥ 25 kg/m^2^) and control group (non-obese with BMI ≤ 25 kg/m^2^). Anthropometric and biochemical characteristics of study subjects are presented in Table 1. In the target group (obese individuals), the levels of total cholesterol (TC, *p* = 0.018) and LDL (*p* = 0.025) were higher when compared with the control group. In contrast, the concentration of HDL was lower (*p* = 0.014) in the obese group. When comparing the level of fasting plasma glucose (FPG) there was no significant difference (*p* = 0.205) between the investigated groups. Significant differences in the prevalence of hypertension, BMI, and T2DM were observed (*p* < 0.001).

PCR amplification and digestion of the amplified fragment with restriction enzyme *HhaI* for adiponectin gene polymorphism rs266729 demonstrated a band of 334 bp for the wild-type (CC) genotype; three bands of 334 bp, 212 bp, and 122 bp were observed for the heterozygous genotype (CG); and two bands of 122 bp and 212 bp were observed for the homozygous genotype (GG). The genotype and allele frequencies of rs266729 polymorphism in the adiponectin gene in obese and non-obese subjects are presented in Table 2. The genotype frequencies observed for the adiponectin gene polymorphism rs266729 were found to be in line with Hardy–Weinberg equilibrium. The findings revealed a significant association of the homozygous GG and heterozygous CG genotypes of the adiponectin gene polymorphism rs266729 with obesity and T2DM. Also, there was a notably higher frequency of the G allele among these subjects (*p* = 0.001). 

The association between obesity, T2DM, biochemical measures, and adiponectin gene rs266729 polymorphism were evaluated by a one-way ANOVA and are presented in Table 3. Notably, the results revealed a significant impact specifically on FPG and HDL levels associated with the rs266729 genotype (*p* < 0.001). There was no significant association between TC and LDL and the three genotypes (CC, CG, GG).

## 3. Discussion

In the present study, the outcomes stemming from the adiponectin gene polymorphism rs266729 analysis revealed a noteworthy association. Individuals carrying the homozygous genotype (GG) exhibited a three-fold increased risk of elevated FPG levels and a higher likelihood of developing T2DM compared with those with the wild-type genotype (CC). Similarly, individuals with the heterozygous (CG) genotype demonstrated a two-fold elevated risk. These observations strongly indicate a potential involvement of the adiponectin gene polymorphism rs266729 in the pathogenesis of T2DM among patients from North Bulgaria. The genotype frequencies observed for the adiponectin gene polymorphism rs266729 were found to be in line with Hardy–Weinberg equilibrium among both control individuals and subjects with obesity and T2DM (*p* > 0.1), aligning with previous findings [32,33,34,35,36]. The identification of genetic variants influencing T2DM remains a primary focus of research aimed at comprehending the underlying mechanisms contributing to the development of this disorder and its associated pathological consequences. The findings from the present study align with the findings reported in Tunisian research [34]; the Kinh Vietnamese population [35]; two Arab populations, specifically in Jordanian subjects [33]; and in a population of Iran [36]. Furthermore, these results are in agreement with the data obtained from French Caucasians [37], Swedish Caucasians [38], and Croatian populations [39]. Conversely, investigations conducted in Omani [40], Taiwanese [41], North Indian [42,43], European [44], Hispanic, and African American [45] populations did not yield significant relationships concerning the investigated SNP. The minor allele frequency of rs266729 observed in the control group (15%) was noted to be lower in comparison with the global minor allele frequency (25%) available from IGSR: The International Genome Sample Resource (https://www.internationalgenome.org/, accessed on 17 January 2026). Moreover, it was also found to be lower in contrast to the frequencies reported in Arab Jordanian populations (22.4%) [33] and Tunisian populations (25.3%) [34]. The observed disparity might be attributed to the relatively small number of subjects in this study, which was conducted during the global SARS-COVID-19 pandemic. The constraints imposed by this extraordinary situation might have influenced the representativeness of the sample, thus potentially impacting the allele frequency estimations.

Understanding the impact of adiponectin gene polymorphism rs266729 on elevated levels of FPG and the development of T2DM requires closer examination of the molecular mechanisms involved. Notably, studies have shed light on the role of specific molecular factors in this process. Specificity protein 1 (Sp1), a transcription factor, was associated with the adiponectin gene promoter. Sp1 is a zinc finger transcription factor that binds to GC-rich motifs within numerous promoters [46]. It was found to enhance gene promoter activity by over-expression [47]. Several studies have revealed that the minor allele G of rs266729 alters the DNA sequence of the Sp1 binding site within the transcriptional elements, which influences the activity of the *ADIPOQ* gene promoter [46,48] because it was shown to influence the DNA-binding activity of the promoter in a 3T3-L1 adipocyte model [49]. Moreover, the G allele of the rs266729 SNP in the adiponectin gene might lead to a loss of the stimulatory Sp1 binding site [48]. This alteration led to a reduction in the transcriptional activity of the adiponectin gene promoter, consequently negatively regulating the expression of the adiponectin gene [46]. This downregulation potentially results in lower plasma concentrations of adiponectin, thereby predisposing individuals to susceptibility to T2DM and obesity [50]. Sp1 activity is involved in various cellular processes, including cell differentiation, cell growth, immune responses, response to DNA damage, and proteolytic processing. Intriguingly, the activity of this protein can act as both an activator and a repressor, significantly influencing diverse cellular functions [51]. Furthermore, multiple studies have reported an association between the minor allele G of rs266729 and reduced plasma adiponectin concentrations [52,53]. However, reduced plasma concentrations of adiponectin might be a result of epigenetic mechanisms, such as DNA hypermethylation [54,55], histone deacetylation [56], and micro-RNAs that suppress adiponectin expression post-translationally [57]. Nevertheless, several investigations have established that alterations in adiponectin levels, whether increased or decreased, correlate with augmented fat burning, reduced fatty acid content, decreased blood glucose levels, and enhanced insulin sensitivity [51,58,59]. Maintaining an appropriate level of adiponectin is crucial for the upkeep of normal tissue and body metabolism.

When analyzing phenotypic data stratified by the genotypes (CC, CG, and GG) of the rs266729 polymorphism, a notable observation emerged, where there were significant differences in HDL and FPG levels in the subjects carrying the three genotypes. Among carriers of the (GG) genotype, the lowest HDL levels were observed, while the (CC) genotype carriers exhibited the highest HDL values. Conversely, the highest FPG levels were noted in individuals with the (GG) genotype, while the lowest values were found in those carrying the CC genotype. Similar findings depicting an inverse relationship between HDL levels and the rs266729 polymorphism were reported by Kaftan and Hussain [32]. Conversely, studies conducted by Zadjali [40] and Sun [60] did not observe such a relationship or association between the rs266729 polymorphism and HDL levels. According to Kaftan and Hussain [32], adiponectin plays a role in influencing HDL levels through various mechanisms. Firstly, adiponectin promotes mitochondrial fatty acid oxidation, consequently leading to a decrease in circulating fatty acid levels. High serum levels of fatty acids inhibit lipoprotein lipase; thus, reducing fatty acid concentrations could alleviate this inhibition. Secondly, adiponectin activates peroxisome proliferator-activated receptor-α (PPAR-α), which upregulates hepatic expression of apoproteins A-I and A-II. This, in turn, facilitates increased hepatic HDL secretion [61]. The current findings present a promising avenue for studying alterations in HDL levels concerning various adiponectin polymorphisms. Understanding these associations could potentially contribute to early prevention and management strategies for cardiovascular diseases.

The study findings revealed a direct correlation between FPG levels and the rs266729 polymorphism. Additionally, an elevated blood pressure was observed within the study group. The existing evidence could suggest the potential presence of insulin resistance in carriers of this polymorphism. When considering the data on FPG levels, changes in HDL levels, and high blood pressure collectively, a plausible role of the rs266729 polymorphism in the development of metabolic syndrome could be suggested.

Although this study evaluated traditional lipid indicators such as TC and HDL, it did not include adipokines (e.g., leptin and resistin) or inflammatory markers such as CRP. Given that adipose tissue dysfunction and chronic low-grade inflammation are central mechanisms in the development of T2DM and considering the regulatory role of *ADIPOQ* in adipokine signaling, future studies incorporating a broader spectrum of metabolic and inflammatory biomarkers would provide a more comprehensive understanding of the complex metabolic disorder network. Notably, significant changes in BMI values were indicated between the control and study groups. However, drawing definitive conclusions is challenging since these changes are not genetically stratified. Several authors have delved into investigating the impact of the rs266729 polymorphism on obesity and have underscored significant variations associated with this polymorphism [37,38,39,40,50,62,63,64]. In contrast, another research study did not observe significant association between obesity and this particular variant [60,65,66,67].

SNP rs266729 in the *ADIPOQ* gene has predictive value for adiponectin levels [43,68,69] and general cardiometabolic risk [70,71], with the G allele often being associated with lower adiponectin and worse metabolic traits. This data reveals that rs266729 has some predictive value for metabolic and cardiovascular risk, but associations are modest and context-dependent. There is emerging evidence that genotypes may influence metabolic improvements after weight loss surgery [72], but robust predictive evidence for pharmacological treatment response is still lacking.

## 4. Materials and Methods

### 4.1. Study Subjects’ Recruitment and Physical and Biochemical Measurements

All individuals were of North Bulgarian origin, and the population was homogeneous regarding ethnic background. The case control study of 156 subjects (*n* = 82 obese and T2DM patients and *n* = 74 non-obese healthy controls according to their BMI) was conducted to assess the associations of SNP rs266729 in the adiponectin (*AdipoQ*) gene with T2DM, lipid profiles, and FPG levels. Written informed consent was provided by all subjects, and a detailed questionary was completed for each participant by a clinical specialist. The procedures implemented in the research followed the declaration of Helsinki, and the research protocol was approved by the Research Ethics Committee of Medical University—Pleven (approval code 355-REC/24.06.2015). The control group included 74 healthy subjects (40 men and 34 women) randomly selected from the general population who attended the hospital for checkup. The inclusion criteria: FPG < 100 mg/dL; no past medical history of T2DM; BMI ≤ 25 kg/m^2^; TC < 200 mg/dL; TG < 150 mg/dL. Subjects were classified as type 2 diabetics if they were on hypoglycemic medications or when their FPG concentrations exceeded 126 mg/dL. Overweight and obesity were defined as a BMI ≥ 25 kg/m^2^ and BMI ≥ 30 kg/m^2^, respectively, based on criteria of the World Health Organization (WHO) classification [73]. The age of the subjects included in the research was between 35 and 85 years, and their residence covered both urban and rural areas. Patients with T2DM were under treatment with oral hypoglycemic agents or insulin. Systolic blood pressure higher than 130 mm Hg and/or diastolic blood pressure higher than 85 mm Hg was defined as hypertension, and these subjects were on antihypertensive medications. At baseline, all study participants were subjected to a thorough screening program that included a detailed assessment of personal and family history, physical examination, determination of anthropometric indices, and measurement of various biochemical parameters. Pregnant women, women up to 3 months postpartum, and those suffering from pathological forms of obesity were excluded from the study by examining medical records, where available.

Venous blood was obtained after overnight fasting. FPG, TC, HDL, and LDL were determined enzymatically using commercially available kits. The blood pressure readings were recorded with the participants sitting after 5 min of resting.

### 4.2. Genotyping Data

Peripheral blood samples were collected from all participants in EDTA anticoagulant tubes. Peripheral blood leucocytes were used for isolation of genomic DNA using column-based DNA extraction (QIAmp DNA Blood Mini Kit, Qiagen, MD, USA). Then, DNA concentration and purity were measured on a NanoDrop^TM^ One spectrophotometer (ThermoScientific, Waltham, MA, USA) by UV absorption at 260 and 280 nm. The purified genomic DNA was diluted to a final working concentration of 5 ng/µL. A polymerase chain reaction-restriction fragment length polymorphism (PCR-RFLP) assay using a qTOWER^3^G (Analytik Jena, Jena, Germany) PCR thermal cycler was implemented for the genotyping of SNP rs266729 in the adiponectin (*AdipoQ*) gene. DNA was amplified using the following primer sequences [74]: forward primer ADPQ-F (22 base pairs and molecular weight 6179.1 Da): 5′-GGTGGACTTGACTTTACTGG-3′; reverse primer ADPQ-R (18 base pairs and molecular weight 5587.7 Da): 5′-TAGAAGCAGCCTGGAGAA-3′ (Metabion International, Planegg, Germany). Primer specificity was tested in a total PCR reaction mixture of 20 µL. The optimal primer annealing temperature was chosen using a gradient PCR ranging from 45 °C to 65 °C in linear increments for 35 cycles. We detected the expected band size only at temperatures ranging from 45 °C to 60.9 °C. Within the tested temperature range, 57.0 °C was the optimal temperature that showed a lack of non-specific amplification. The PCR amplification was carried out in a reaction mixture of 20 µL final volume by using 5 ng/µL genomic DNA, 0.3 µM of each primer, 200 µM dNTP (ThermoScientific, Waltam, MA, USA), 1.5 µM MgCl_2_, 0.5 U of Taq DNA polymerase (ThermoScientific, Waltam, MA, USA), and 10 µM Tris-HCl (pH 7.4). The PCR consisted of initial denaturation for 5 min at 94 °C, followed by 35 cycles of denaturation at 94 °C for 30 s, annealing at 57 °C for 45 s, an extension at 72 °C for 45 s, and with a final extension at 72 °C for 5 min.

PCR product 334 bp for *AdipoQ* was visualized on 2.5% agarose gel containing ethidium bromide. The polymorphism was typed by using aliquots of the PCR product digested overnight at 37 °C with *Hhal* restriction enzyme (ThermoScientific, Waltam, MA, USA). The restriction products for *AdipoQ* rs266729 polymorphism with respective size (212 bp and 121 bp) were visualized by using 2.5% agarose gel electrophoresis stained with ethidium bromide.

### 4.3. Statistical Analysis

The statistical analysis used to compare phenotypic data between control and study groups was performed using IBM SPSS Statistics for Windows, version 29 (IBM Corp., Armonk, NY, USA). The comparison between obese (target) and non-obese (control) subjects was done by an unpaired *t*-test. The distribution of genotypes and the frequency of the alleles were determined, and a *chi*-square test was used for comparison. A one-way analysis of variance test (ANOVA) was used to check whether there is difference between the anthropometric and biochemical parameters among the subjects with different genotypes. Genotype numbers were determined manually from the allelic discrimination plots, and allele frequencies were determined from the genotype frequencies. All *p*-values < 0.05 were considered significant.

## 5. Conclusions

The adiponectin gene polymorphism rs266729 has been linked to elevated FPG levels and an increased risk of developing T2DM in the Northern Bulgarian population. Individuals carrying the homozygous genotype (GG) exhibit the highest risk of developing T2DM, while carriers of the (CG) genotype also demonstrate an increased susceptibility. Moreover, carriers of the rs266729 polymorphism in the adiponectin gene display a higher prevalence of high blood pressure and obesity. This genetic variant appears to contribute to an elevated risk of developing cardiovascular diseases and metabolic syndrome, potentially influencing HDL levels and glucose regulation.

## Figures and Tables

**Table 1:** Phenotypic data (anthropometric and biochemical) of obese (target) and non-obese (control) subjects.

Phenotypic Data	Obese and T2DM (*n* = 82)	Non-Obese (*n* = 74)	*p*-Value
Age (years)	69.15 ± 0.87	72.35 ± 0.84	0.009
BMI (kg/m^2^)	34.23 ± 0.21	22.42 ± 0.15	<0.001
TC (mmol/L)	4.55 ± 0.15	5.05 ± 0.15	0.018
HDL (mmol/L)	1.22 ± 0.05	1.38 ± 0.03	0.014
LDL (mmol/L)	2.61 ± 0.12	3.02 ± 0.13	0.025
FPG (mmol/L)	6.58 ± 0.26	6.16 ± 0.20	0.205
Hypertension (%)	47.4	21.8	<0.001
Diabetes Mellitus (%)	26.3	10.9	<0.001

Number of individuals (*n*) is in parentheses. Student’s *t*-test was used to compare continuous variables, and the results are presented as the mean ± SD. Categorical variables were compared by a *chi*-square test. *p* < 0.05 was considered statistically significant. BMI, body mass index; TC, total cholesterol; HDL, high-density lipoprotein cholesterol; LDL, low-density lipoprotein cholesterol; FPG, fasting plasma glucose.

**Table 2:** Genotype distribution and allele frequency of rs266729 polymorphism and association of these variants with obesity and T2DM in the study subjects.

Genotype rs266729 (C/G)	Control *n* = 74 (%)	Obese, T2DM *n* = 82	Unjustified OR (95% CI) *p*-Value	Adjusted OR for Age and BMI (95% CI) *p*-Value
CC	56 (75.67%)	46 (56.1%)		
CG	14 (18.92%)	23 (28.05%)	2.40 (1.4–4.18) 0.002	2.30 (1.40–4.22) 0.001
GG	4 (5.41%)	13 (15.85%)	3.55 (1.30–10.70) 0.01	3.62 (1.28–10.70) 0.01
Frequency of G allele	22 (15%)	49 (30%)	2.39 (1.50–3.72) 0.001	

Data are presented as number of cases (*n*) with frequencies in parentheses. OR, odds ratio; CI, confidence interval; T2DM, type 2 diabetes mellitus.

**Table 3:** Association between the metabolic parameters in the subjects with obesity (BMI > 25 kg/m^2^) and T2DM and the rs266729 polymorphism in the adiponectin gene.

Parameter	Genotype	Mean ± SE	95% CI for Mean	ANOVA *p*-Value
TC (mmol/L)	CC	4.64 ± 0.52	3.53–5.76	0.618
	CG	4.64 ± 0.20	4.24–5.04	
	GG	4.31 ± 0.19	3.91–4.70	
HDL (mmol/L)	CC	1.58 ± 0.16	1.33–1.94	<0.001
	CG	1.08 ± 0.07	0.98–1.18	
	GG	0.70 ± 0.07	0.51–0.23	
LDL (mmol/L)	CC	2.83 ± 0.45	1.86–3.79	0.444
	CG	2.66 ± 0.16	2.35–2.98	
	GG	2.38 ± 0.15	2.07–2.69	
FPG (mmol/L)	CC	4.53 ± 0.17	4.16–4.89	<0.001
	CG	5.92 ± 0.15	5.62–6.22	
	GG	9.20 ± 0.54	8.08–10.32	

Data are presented as the mean ± SD. TC, total cholesterol; HDL, high-density lipoprotein cholesterol; LDL, low-density lipoprotein cholesterol; FPG, fasting plasma glucose; CI, confidence interval.

## Data Availability

The original contributions presented in this study are included in the article. Further inquiries can be directed to the corresponding author.
